# Viral protein Nef is detected in plasma of half of HIV-infected adults with undetectable plasma HIV RNA

**DOI:** 10.1371/journal.pone.0191613

**Published:** 2018-01-24

**Authors:** Jana Ferdin, Katja Goričar, Vita Dolžan, Ana Plemenitaš, Jeffrey N. Martin, Boris M. Peterlin, Steven G. Deeks, Metka Lenassi

**Affiliations:** 1 Institute of Biochemistry, Faculty of Medicine, University of Ljubljana, Ljubljana, Slovenia; 2 Department of Epidemiology & Biostatistics, University of California, San Francisco, San Francisco, California, United States of America; 3 Department of Medicine, Microbiology and Immunology, University of California, San Francisco, San Francisco, California, United States of America; University Hospital Tuebingen, GERMANY

## Abstract

**Objective:**

To address the role of translationally active HIV reservoir in chronic inflammation and non-AIDS related disorders, we first need a simple and accurate assay to evaluate viral protein expression in virally suppressed subjects.

**Design:**

We optimized an HIV Nef enzyme-linked immunosorbent assay (ELISA) and used it to quantify plasma Nef levels as an indicator of the leaky HIV reservoir in an HIV-infected cohort.

**Methods:**

This study accessed 134 plasma samples from a well-characterized cohort study of HIV-infected and uninfected adults in San Francisco (the SCOPE cohort). We optimized an ELISA for detection of plasma Nef in HIV-negative subjects and HIV-infected non-controllers, and evaluated its utility to quantify plasma Nef levels in a cross-sectional study of ART-suppressed and elite controller HIV-infected subjects.

**Results:**

Here, we describe the performance of an optimized HIV Nef ELISA. When we applied this assay to the study cohort we found that plasma Nef levels were correlated with plasma HIV RNA levels in untreated disease. However, we were able to detect Nef in plasma of approximately half of subjects on ART or with elite control, despite the lack of detectable plasma HIV RNA levels using standard assays. Plasma Nef levels were not consistently associated with CD4+ T-cell count, CD8+ T-cell count, self-reported nadir CD4+ T-cell count or the CD4+/CD8+ T-cell ratio in HIV-infected subjects.

**Conclusion:**

Since plasma HIV RNA levels are undetectable in virally suppressed subjects, it is reasonable to assume that viral protein expression in leaky reservoir, and not plasma virions, is the source of Nef accumulating in plasma. To examine this further, improvements of the assay sensitivity, by lowering the background through improvements in the quality of Nef antibodies, and detailed characterization of the HIV reservoirs are needed.

## Introduction

Effective antiretroviral therapy (ART) reduces plasma HIV RNA levels to below the limit of quantification (< 20 to 50 RNA copies/ml) in the vast majority of treated HIV-infected individuals [1], still the virus persists indefinitely [2]. Also, ART does not fully restore immune function and health, supporting chronic inflammatory environment, which has been associated with an increased risk for a variety of non-AIDS-related disorders. Other contributing factors are a higher prevalence of traditional risk factors (smoking, alcohol, substance abuse) and the direct toxic effects of ART drugs [2]. The degree to which HIV persistence contributes to chronic inflammation and non-AIDS morbidity remains largely unknown.

Much effort has been made towards developing assays to measure HIV reservoirs but each approach has limitations [3, 4]. The quantitative viral outgrowth assay is laborious, expensive and only provides a minimal estimate of the true reservoir size [5]. In contrast, PCR-detection of HIV DNA is more practical but cannot readily distinguish defective from intact genomes and hence overestimates the true reservoir size [6]. Reverse-transcription PCR-detection of intracellular and virion HIV RNA provides an (over)estimate of the more transcriptionally active reservoir [3, 4]. Fluorescent *in situ* hybridization connected to flow cytometry enables single-cell characterization of HIV RNA and proteins in re-activated HIV reservoirs, but assay sensitivity may be limiting [7, 8]. Importantly, viral proteins Nef and Env were detected in plasma of some virally suppressed subjects, indicating a need for evaluating the translationally active reservoir [9, 10].

Nef is a crucial determinant of viral pathogenesis and disease progression [11]. It is one of the early, completely spliced HIV transcripts. Hence it does not require viral protein Rev for export to cytosol, where Nef is translated to high protein levels [12]. Nef affects diverse processes inside the HIV-infected cell, but is also released extracellularly through incorporation into virions [13]. Alternatively, Nef was shown to induce its own release from HIV-infected cells with exosomes (extracellular vesicles of endosomal origin) [14–18], although some concerns were raised about the exosome-bound nature of extracellular Nef [19]. Still, Nef-containing exosomes were detected in the plasma of suppressed HIV-infected individuals [9, 17]. Altogether, plasma Nef levels may serve as a good indicator of viral protein expression in virally suppressed subjects.

To address the role of translationally active HIV reservoir in chronic inflammation and non-AIDS-related disorders, we first need a simple and accurate assay to evaluate viral protein expression in virally suppressed subjects. To this end, we optimized an ELISA to determine Nef concentrations in plasma as an indicator of the leaky HIV reservoir. We used this assay to quantify plasma Nef levels in a cross-sectional study of ART-suppressed and elite controllers and examined Nef association with subject’s clinical characteristics.

## Materials and methods

### Study subject

This study accessed 134 plasma samples from a well-characterized cohort study of HIV-infected and uninfected adults in San Francisco (the SCOPE cohort). All subjects provided written informed consent and the parent study was approved by the UCSF Committee on Human Research. In brief, whole blood samples were collected into commercially available EDTA-treated tubes, processed by low-speed centrifugation (2000*g* for 15 min) within 2 h, and the supernatants were aliquoted, frozen and stored at -70°C. All subjects were characterized with respect to age, gender, ethnicity, HIV status (serostatus, blood CD4+ and CD8+ T-cell counts, plasma HIV RNA concentrations) and ART regimen. Specifically, plasma HIV RNA concentrations were detected using one of the following commercially available assays: the Roche COBAS® AmpliPrep/COBAS® TaqMan® HIV-1 Test (detection limit 20 RNA copies/ml), the Abbott RealTime HIV-1 (40 RNA copies/ml) or the Bayer Versant HIV-1 RNA Assay (75 RNA copies/ml).

The study subjects were stratified into four groups: (i) HIV uninfected subjects (HIV-negative); (ii) ART naive HIV-infected subjects with high level viremia (non-controllers); (iii) HIV-infected subjects with undetectable plasma HIV RNA for at least the last six months while taking a stable ART (ART-suppressed); and (iv) untreated HIV-infected subjects with undetectable plasma HIV RNA levels for at least the last twelve months (elite controllers).

### HIV Nef enzyme-linked immunosorbent assay

**Fig 1:**
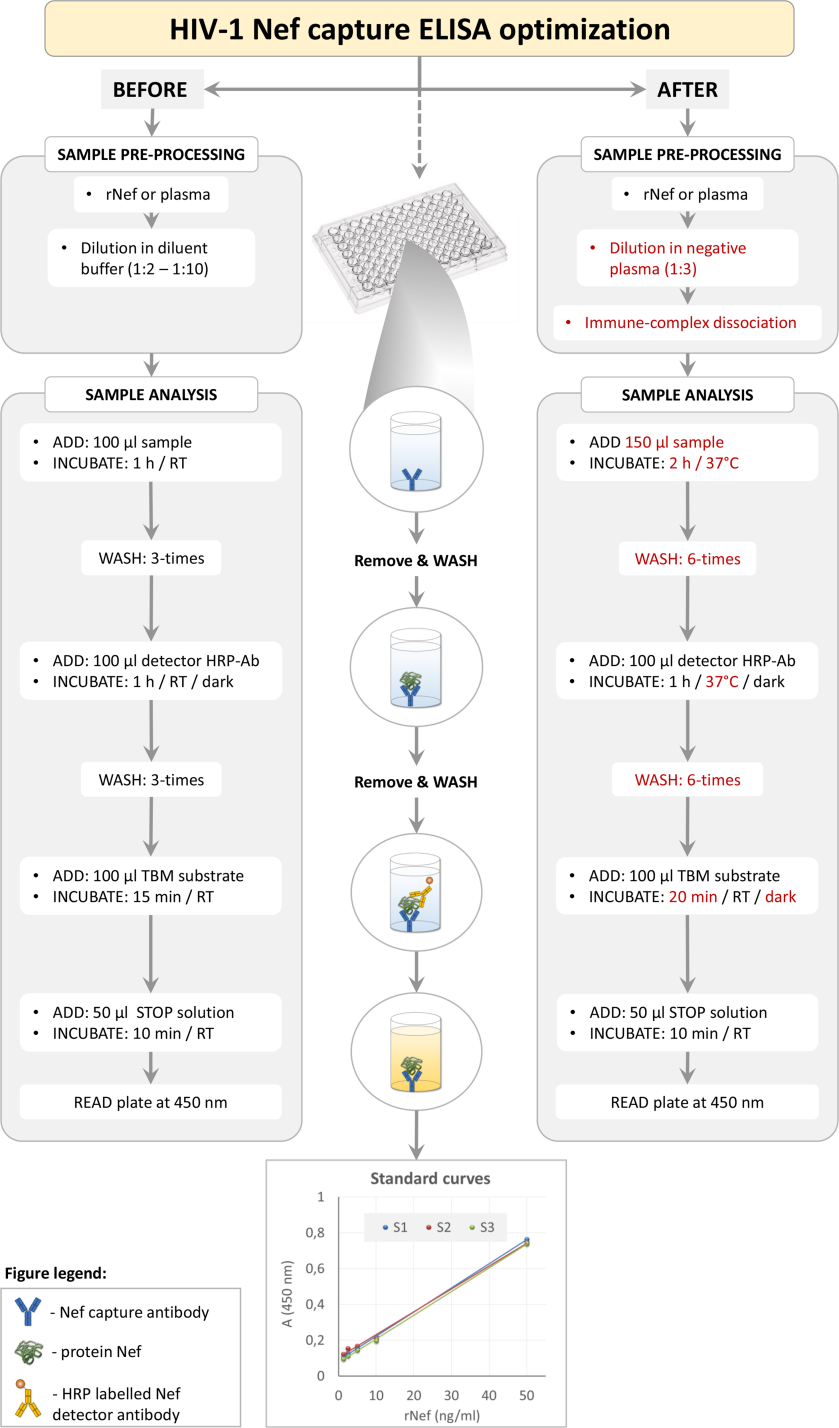
Schematic representation of HIV Nef ELISA optimization. Key assay optimizations are highlighted by the red text. Three linear standard curves of serially diluted Nef in negative serum (1.25 ng/ml to 50 ng/ml), designated as S1, S2 and S3, are shown as assay readouts. rNef, recombinant Nef; RT, room temperature; HRP-Ab, horseradish peroxidase-conjugated antibody; TBM, 3,3',5,5'-tetramethylbenzidine; A, absorbance. For details, see Materials and methods.

To determine plasma Nef concentration we optimized the commercially available Nef capture ELISA (ImmunoDx Ilc., MA), which showed extremely high background for complex plasma samples when following manufacturers’ instructions. We modified the sample pre-processing step and the assay volumes, incubation times, temperatures, and washing steps (Fig 1). Specifically, plasma samples were first slowly defrosted on ice and centrifuged at 2000*g* for 30 min to remove precipitated lipids. Next, 50 μl of each sample was lysed by the addition of 5% TritonX-100 to 1% of final volume and further processed for immune complex dissociation. Briefly, 45 μl of 0.33N HCl was added to the sample and incubated for 1 h at 37°C, after which 45 μl of 0.33N NaOH was added and incubated for 20 min at room temperature. Processed sample (total volume 150 μl) was transferred to ELISA plates coated with murine anti-Nef monoclonal antibodies and incubated for 2 h at 37°C. Plates were washed six times with wash buffer, after which 100 μl of murine anti-Nef-HRP-labeled detector antibody was added to each well and incubated for 1 h at 37°C in the dark. According to the manufacturer, the above mentioned capture and detector antibodies bind to the opposite (N- and C- terminal) parts of protein Nef. Following, plates were washed six times before 100 μl of 3,3',5,5'-tetramethylbenzidine (TMB) substrate was added for colorimetric detection and incubated in the dark at room temperature for 20 min. To terminate the reaction, 50 μl of stop solution was added and after 10 min the absorbance was measured at 450 nm using Synergy 2 Multi-Mode Reader (Bio-Tek, Germany). Nef concentration in plasma samples was calculated from the linear standard curve of serially diluted recombinant Nef in negative serum (1.25 ng/ml to 50 ng/ml), which were processed as described above for the tested plasma samples. All measurements were performed in triplicates. ELISA was performed blinded regarding individuals clinical status.

### Immunoblotting

Immunoblotting is not an appropriate method for directly testing for presence of specific proteins in plasma, as abundant plasma proteins like albumin overshadow the remainder of the plasma proteins. Therefore, viruses and/or extracellular vesicles were first enriched from 3 ml of plasma samples as described by Lee et al. [20], and later lysed, separated by 12% SDS-PAGE and transferred to PVDF membrane (Millipore) as described previously [18]. Rabbit polyclonal antibodies to HIV-1 Nef (NIH AIDS Reagent Program, 2949) or extracellular vesicle marker CD63 (sc-15363), and mouse monoclonal antibodies to HIV-1 p24/gag (ab9071, Abcam) or extracellular vesicle marker flotillin (610820, BD Bioscience, NJ, USA), were used as primary antibodies; and appropriate HRP-conjugated anti-rabbit or anti-mouse (Jackson Immunoresearch, PA, USA) were used as secondary antibodies. Membranes were developed by SuperSignal West Pico Chemiluminescent Substrate (Thermo Scientific).

### Statistical analysis

All analyses were performed using IBM SPSS Statistics version 19.0 (IBM Corporation, NY). Median and interquartile range were used to describe continuous variables. Receiver operating characteristic (ROC) curve was used to determine the background of the HIV Nef ELISA in plasma based on 100% specificity (no false positives) when comparing the absorbance values of HIV-negative samples and the absorbance values in non-controllers. Nonparametric Kruskal-Wallis test or Spearman’s rho correlation coefficient were used to compare differences or calculate correlations for continuous variables. Fisher’s exact test was used for comparisons of categorical variables. *P* values less than 0.05 were considered statistically significant.

## Results

Clinical characteristics of study participants (N = 134) are presented in Table 1. We studied four groups: HIV-negative (n = 26), non-controllers (n = 28), ART-suppressed (n = 38) and elite controllers (n = 42). As expected, the distributions of CD4+ T-cells, CD8+ T-cells, CD4+/CD8+ T-cell ratios and nadir CD4+ T-cells differed significantly among the four groups (*P*<0.001 for all).

**Table 1:** Clinical characteristics of study participants.

Clinical characteristics*		All (N = 134)	HIV-negative (n = 26)	Non-controllers (n = 28)	ART-suppressed^¶^ (n = 38)	Elite controllers (n = 42)	*P*^#^
**Gender**	**male**	109	25	25	31	28	0.001^$^
	**female**	20	1	0	7	12	
	**others^&^**	5	0	3	0	2	
**Age (years);** **median (min—max)**		49 (26–73)	46 (26–71)	47 (29–71)	51 (28–73)	50 (32–72)	0.111
**Plasma HIV RNA in infected individuals (copies/ml)**		/	<40^•^	30,837 (19,526–80,149)	<75^•^	<75^•^	/
**CD4+ T-cells (cells/mm^3^)**		685 (460–974)	792 (686–1,020)	393 (254–586)	524 (416–718)	952 (680–1,125)	<0.001
**CD8+ T-cells (cells/mm^3^)**		756 (530–1,069)	446 (353–616)	974 (762–1,670)	768 (581–1,068)	820 (550–1,069)	<0.001
**CD4+ / CD8+ T-cell ratio**		0.83 (0.50–1.51)	1.75 (1.31–2.36)	0.40 (0.24–0.50)	0.63 (0.47–0.88)	1.18 (0.84–1.58)	<0.001
**Self-reported nadir CD4+ T-cells (cells/mm^3^)^†^**			/	400 (240–570)	97 (27–253)	600 (395–800)	<0.001
**Individuals with detectable plasma Nef (n)**		63	0	23	18	22	<0.001^$^
**Detectable plasma Nef level (ng/ml)**		10.23 (7.01–11.98)	/	11.63 (9.04–13.26)	8.25 (6.83–10.95)	8.78 (6.52–11.66)	0.037

* All values represent median (25%–75%) if not indicated otherwise.

^¶^ ART regimen: 18 (47.4%) received protease inhibitor (PI)-based therapy, 11 (28.9%) received non-nucleoside reverse transcriptase inhibitor (NNRTI)-based therapy, 5 (13.2%) received therapy that included both, PI and NNRTI, and 4 (10.5%) were on regimens that did not contain either. Median duration of ART (min—max) was 2244 days (184–6809).

^#^ Statistical comparison of subgroups using nonparametric Kruskal-Wallis test if not indicated otherwise.

^$^ Calculated using Fisher exact test.

^&^ Presenting intersex or male to female transition individuals.

^•^Three different tests were used for plasma HIV RNA detection with different limits of detection: in 23 out of 108 tested individuals the Roche COBAS® AmpliPrep/COBAS® TaqMan® HIV-1 Test (detection limit 20 RNA copies/ml), in 67/108 individuals the Abbott RealTime HIV-1 (40 RNA copies/ml) and in 18/108 individuals the Bayer Versant HIV-1 RNA Assay (75 RNA copies/ml) were used. The presented value is thus the limit of detection of the least sensitive assay.

^†^ Data was not available for one non-controller, two ART-suppressed and six elite controllers.

**Fig 2:**
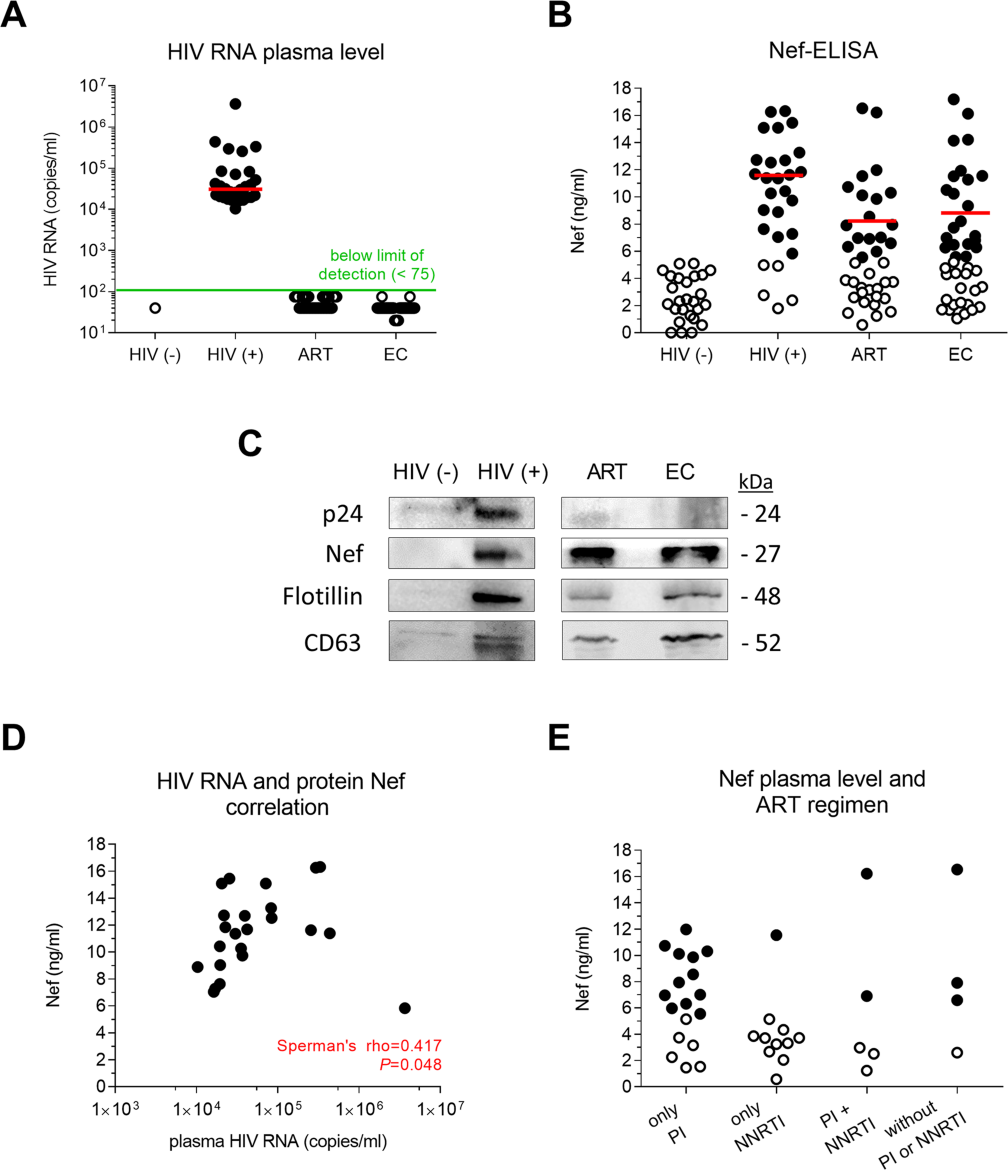
Viral protein Nef was detected in plasma of approximately half of aviremic HIV-infected subjects. Red lines represent the median value. (**A**) Real-time PCR detection of plasma HIV RNA levels in study participants. Full circles represent exact plasma HIV RNA levels, while empty circles represent the limit of detection of the assay used to measure individual’s plasma HIV RNA. (**B**) Detection of plasma Nef levels in study participants using optimized HIV Nef ELISA. Full circles represent measured plasma Nef levels, while empty circles represent background values. The background level of the HIV Nef ELISA, determined by ROC curve analysis, was 5.46 ng/ml. (**C**) Immunoblot of lysed viruses and/or extracellular vesicles enriched from representative plasma samples of the HIV-negative, non-controller, ART-suppressed and elite controller groups. Antibodies directed against HIV proteins p24 and Nef, and against extracellular vesicle markers flotillin and CD63, were used. (**D**) Correlation between plasma HIV RNA and detectable Nef levels in non-controllers. (**E**) Plasma Nef levels in aviremic ART-suppressed subjects treated with various ART regimens. Full circles represent measured plasma Nef levels, while empty circles represent assay background values. HIV (-), HIV-negative; HIV (+), non-controller; ART, ART-suppressed; EC, elite controller; PI, protease inhibitor-based therapy; NNRTI, non-nucleoside reverse transcriptase inhibitor-based therapy.

We first optimized the HIV Nef ELISA for detection of Nef diluted in complex plasma samples as described in Fig 1. The reproducibility of the assay was shown by repeated measurements of serially diluted recombinant Nef (1.25 ng/ml to 50 ng/ml), which gave comparable standard curves (Fig 1). Next, using measured plasma Nef levels in HIV-negative subjects (true negatives) and HIV-infected non-controllers (true positives; Fig 2A and 2B; Table 1) as input for ROC curve analysis, the background level of the HIV Nef ELISA based on 100% specificity was determined as 5.46 ng/ml. Therefore plasma Nef concentrations above 5.46 ng/ml were defined as detectable plasma Nef.

Twenty-three (82.1%) non-controllers had detectable plasma Nef levels with median concentration of 11.63 ng/ml. The presence of Nef in plasma of non-controllers was additionally supported by immunodetection of Nef in a representative sample, enriched for viruses (immunodetection of p24) and extracellular vesicles (immunodetection of flotillin, CD63) (Fig 2C). In non-controllers plasma Nef levels correlated positively with plasma HIV RNA levels (r = 0.417, *P* = 0.048; Fig 2D and Table 1). No correlation was observed with CD4+ T-cells, CD8+ T-cells, self-reported nadir CD4+ T-cells and CD4+/CD8+ T-cell ratio (all *P*>0.05).

Nef was also detected in plasma of 47.4% (18/38) of ART-suppressed subjects, with a median concentration of 8.25 ng/ml, and in 52.4% (22/42) of elite controllers, with a median concentration of 8.78 ng/ml (Fig 2B and Table 1). This was supported by immunodetection of Nef in representative plasma samples of ART-suppressed subjects and elite controllers, enriched for extracellular vesicles (immunodetection of flotillin, CD63) (Fig 2C). In both groups, subjects with detectable plasma Nef did not differ significantly from subjects with undetectable plasma Nef with regard to age, CD4+ T-cells, CD8+ T-cells, self-reported nadir CD4+ T-cells and CD4+/CD8+ T-cell ratio (all *P*>0.05). In ART-suppressed subjects plasma Nef was more commonly detected in those on PI-based therapies (60.9%, 14/23) than NNRTI-based therapies (18.8%, 3/16) (Table 1 and Fig 2E).

## Discussion

Quantification of Nef levels in plasma of aviremic HIV-infected adults may serve as a measure of viral protein expression in leaky HIV reservoir. Here, we describe the performance of an optimized HIV Nef ELISA, verified by immunoblotting. When we applied this assay to a large cohort of HIV-uninfected and HIV-infected adults we found that plasma Nef levels correlated with plasma HIV RNA levels in untreated disease. However, we were able to detect Nef in plasma of approximately half of subjects on ART or with elite control, despite the lack of detectable plasma HIV RNA levels using standard diagnostic assays.

Nef is incorporated into HIV virions in small quantities, where it stably associates with the HIV capsid [13, 18]. This explains our observation that plasma Nef levels correlate with plasma HIV RNA levels in non-controllers. Although there was a reasonable association between Nef levels and HIV RNA levels in untreated subjects, 5 of 28 non-controllers lacked detectable Nef. Similarly, Fujii et al. [21] detected Nef in plasma of only 27 out of 32 HIV-1 seropositive subjects. The underlying reason may be the intra- and inter-subtype variability of Nef sequence that could affect Nef virion incorporation [22, 23] and recognition by ELISA capture and detector antibodies. The study by Mann et al. [24] namely showed that 16 amino acids occur at 10 unique Nef codons in subtype B, the dominant HIV subtype in the Americas, Western Europe and Australasia. Even greater variability was shown for Nef from different HIV subtypes, as 84% sequence identity was observed in 186 strains tested [25]. Several of these variable sites are located to N- and C-terminal parts of Nef, which serve as epitopes recognized by antibodies.

Further work will be needed to define the source of plasma Nef in well-controlled HIV infection, since plasma HIV RNA levels are undetectable, thereby excluding HIV virions as the possible source. Although not specifically tested in this study, it is reasonable to assume that viral protein expression in leaky reservoirs, including cells harboring replication competent or defective HIV proviruses, are the source of Nef accumulating in plasma of virally suppressed individuals. Several recent studies support this observation. First, Nef is an early HIV transcript, which does not need other viral proteins for high expression in the host cell [12]. Second, over 93% of all proviruses are defective [6], but can produce novel HIV proteins [26]. Third, Nef was shown to facilitate its own export with exosomes from Nef-expressing or HIV-infected T-cells *in vitro* [14, 16, 17], although Luo et al. [19] questioned that. Fourth, as also shown in this study, Nef [21] and Nef-containing extracellular vesicles [9, 17] were detected in plasma of HIV-infected individuals [9].

There was no consistent association between plasma Nef levels and clinical characteristics in HIV-infected subjects. In contrary, Lee et al. showed inverse relationship between plasma Nef levels and CD4+ and CD8+ T-cell counts in a cohort of 17 aviremic and 16 viremic HIV-infected subjects [9]. Reasons for this discrepancy could be a greater variability of subjects clinical characteristics in a relatively small study cohort (n = 33) and the use of a semi-quantitative method (western blot analysis) for plasma Nef detection in the study by Lee et al. [9]. Previous studies suggest that PI-based therapies may permit a low-level viral replication [27, 28], still the association between plasma Nef and ART regimen shown in this study may be confounded by the influence of the time of initiation and adherence to ART on the size of HIV reservoirs.

In conclusion, we showed that a simple ELISA can detect plasma Nef in half of HIV-infected subjects with undetectable plasma HIV RNA. To further evaluate plasma Nef level as a quantitative marker of translationally active HIV reservoir in aviremic individuals, improvements of the assay sensitivity, by lowering the background through improvements in the quality of Nef antibodies, and detailed characterization of the HIV reservoirs in the study cohort are needed.
